# Red Mold Rice Mitigates Oral Carcinogenesis in 7,12-Dimethyl-1,2-Benz[a]anthracene-Induced Oral Carcinogenesis in Hamster

**DOI:** 10.1093/ecam/nep215

**Published:** 2011-05-04

**Authors:** Ruei-Lan Tsai, Bing-Ying Ho, Tzu-Ming Pan

**Affiliations:** ^1^R&D Division, Sunway Biotechnology Company Limited, Taipei, Taiwan; ^2^Institute of Microbiology and Biochemistry, College of Life Science, National Taiwan University, Taipei, Taiwan

## Abstract

The prevalence of oral tumor has exponentially increased in recent years; however, the effective therapies or prevention strategies are not sufficient. Red mold rice is a traditional Chinese food, and several reports have demonstrated that red mold rice had an anti-tumor effect. However, the possible anti-tumor mechanisms of the red mold rice are unclear. In this study, we examined the anti-tumor effect of red mold rice on 7,12-dimethyl-1,2-benz[a]anthracene (DMBA)-induced oral tumor in hamster. The ethanol extract of red mold rice (RMRE) treatment significantly decreases the levels of DMBA-induced reactive oxygen species, nitro oxide and prostaglandin E_2_ than those of the lovastatin-treated group (*P* < .001). Moreover, RMRE decreases the formation of oral tumor induced by DMBA. Monacolin K, monascin, ankaflavin or other red mold rice metabolites had been reported to decrease inflammation and oxidative stress and exerted anti-tumor effects. Therefore, we evaluated the anti-inflammation and anti-oxidative stress effects of monacolin K, monascin, ankaflavin and citrinin in lipopolysaccharide-treated RAW264.7 cells. We found that RMRE reduced the LPS-induced nitrite levels in RAW264.7 cells better than monacolin K, monascin, ankaflavin or citrinin (*P* < .05).

## 1. Introduction

The incidence and mortality rates of head and neck tumor have been increasing in recent years, especially oral tumor [1]. Despite improvement in surgery, radiotherapy and chemotherapy, the 5-year survival rate of head and neck cancers have improved marginally [2]. Therefore, it is important to renew in chemoprevention as a means of reducing the incidence and mortality of these cancers.

*Monascus*-fermented rice, known as red mold rice, is a common food item found in China, used to enhance the color and flavor of food, as well as a traditional medicine for digestive and vascular functions [3, 4]. Currently, red mold rice is regarded as a popular health food for hypolipidemic treatment in Asia and the United States and several components of red mold rice have been identified. Monacolins, a group of 3-hydroxzy-3-methyglutaryl-coenzyme A reductase inhibitors with characteristics identical to those of statins, are the functional ingredients of red mold rice with hypolipidemic ability [5, 6]. In addition, several secondary metabolites have also been identified, such as azaphilone pigments (e.g., monascin, ankaflavin, rubropunctatin, monascorburin, ruborpunctamine and monascorburamine) exhibited anti-inflammatory and anti-tumor activities or a group of antioxidants, including dimerumic acid, tannin and phenol [7–12].

Oxidative stress due to high flux of oxidants has been implicated in the pathogenesis of several cancers, including oral cancer [13]. Over-production of reactive oxygen species (ROS) has been well documented in betel quid and tobacco chewers [14]. ROS-mediated oxidative damage results in deoxyribonucleic acid damage, and thereby contributes to mutagenesis and carcinogenesis [15]. 7,12-Dimethyl-1,2-benz[a]anthracene (DMBA) is a potent carcinogen. It has been suggested that DMBA, on metabolic activation, induces cancer through an oxidative mediated genotoxicity by incorporating diolepoxide and other ROS into DNA [16]. The DMBA-induced buccal-pouch mucosa carcinogenesis in hamster is one of the most extensively used models for oral carcinogenesis investigation since it has many morphological and histological similarities with human oral carcinoma [17].

In this study, we investigated the ability of the ethanol extract of red mold rice (RMRE) in DMBA-induced hamster buccal pouch squamous-cell carcinogenesis and we also clarified the possible anti-tumor effect of RMRE.

## 2. Methods

### 2.1. Chemical and Reagents

Lovastatin was obtained from Standard Chem. & Pharm. Co. Ltd (Tainan, Taiwan) and celecoxib was purchased from Pfizer (NY, USA). 7,12-Dimethyl-1,2-benz[a]anthracene (DMBA), sodium nitrite, lipopolysaccharide (LPS) and nitro blue tetrazolium (NBT) were purchased from Sigma-Aldrich. (St. Louis, MO, USA). Prostaglandin E_2_ immunoassay kit was purchased from Cayman Chemical Co. (Ann Arbor, MI, USA). The NO assay kit was purchased from R&D (Minneapolis, MN, USA).

### 2.2. Preparation of Ethanol Extraction of Red Mold Rice

The long-grain rice was purchased from a local supermarket in Taiwan to be used for red mold rice production under solid-state cultivation. *Monascus purpureus* NTU 301 was used to prepare red mold rice via the method in previous study [6]. After fermentation, the crushed and dried red mold rice (1 kg) was further extracted by ethanol at 50°C for 3 days. The extracts were concentrated by vaccum filtration device and dried by lyophilization. The key components in ethanol extraction of red mold rice (RMRE) contain 5.092 mg monacolin K per g powder, 0.028 mg citrinin per g powder, 12.844 mg per g monascin and 4.124 mg ankaflavin per g powder. The methods for analyzing the key components in RMRE are cited from these papers [18, 19]. The dried ethanol extract of *M. purpureus* NTU 301-fermented red mold rice was resolved with ethanol at a final concentration of 50 mg mL^−1^ and stored at 4°C.

### 2.3. DMBA-Induced Oral Tumor Model

**Figure 1:**
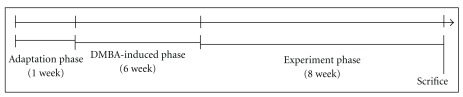
The flowchart of oral tumor induction and drug treatment. To induce oral tumor formation (DMBA-induced phase), 0.5% DMBA solution was prepared in mineral oil and applied to the entire mucosal surface of the right buccal pouch for 3 times/week. After 6 weeks, 6% celecoxib, RMRE (22.7 mg kg^−1^) or lovastatin (0.15 mg kg^−1^) was administrated with mineral oil to DMBA-induced oral tumor hamsters (experiment phase).

Five-week-old male Syrian golden hamsters were purchased from National Laboratory Animal Center (Taipei, Taiwan). Hamsters were housed in a temperature at 23 ± 2°C, 50 ± 10% humidity and kept on a 12 : 12 light : dark cycle. The animal procedures were performed according to the Guide for the Care and Use of Laboratory Animals of the National Institutes of Health, as well as the guidelines of the Animal Welfare Act. The model of DMBA-induced hamster cheek pouch carcinogenesis was modified from Salley [20]. As shown Figure 1, the animals were treated by painting a buccal pouch three times a day for 6 weeks with a 0.5% solution of DMBA dissolved in mineral oil. The dosage of RMRE was calculated in accordance with Boyd's formula of body surface area as recommended by the Food and Drug Administration (FDA) [6]. A dose of 22.7 mg RMRE per kg body weight was fed in hamster that is recommended to the supplement of the daily diet at 200 mg for an adult with a weight of 65 kg and a height of 170 cm. And lovastatin group provided 0.15 mg lovastatin per kg body weight that is equal to monacolin K in RMRE group. Each animal of positive control group was treated with 100 *μ*L 6% celecoxib following the method described in previous study [21, 22]. Animals were killed after 14 weeks and the tumor volume and burden were determined as follows [23].

Tumor volume was measured using the formula *V* = 4/3 (*D*_1_/2)(*D*_2_/2)(*D*_3_/2), where *D*_1_, *D*_2_ and *D*_3_ are the three diameter (mm) of the tumor:

(1) $$\begin{array}{cc} & \text{Tumor}\;\text{burden}\\ & \;=\text{Total}\;\text{numbers}\;\text{of}\;\text{tumors}\;\times \text{Mean}\;\text{volume}\;\left(4/3\pi {\text{r}}^{3}\right). \end{array}$$

Results are presented as mean ± SD.

### 2.4. Determination of ROS, NO_2_^−^/NO_3_^−^ and PGE Levels in Tissue Homogenate

In the measurement of ROS, 100 *μ*L homogenates of tissue were added to 96-well plates, and reacted with 25 *μ*L NBT (10 mg mL^−1^) at 37°C for 2 h and measured by absorbance at 600 nm [24]. In the measurement of NO_2_^−^/NO_3_^−^ or PGE_2_, 100 *μ*L homogenates were filtered by 10 kD filter, and then measured by the NO kit or a prostaglandin E_2_ immunoassay kit [25].

### 2.5. Cell Culture

Murine macrophage cell line, RAW264.7, was obtained from the Bioresource Collection and Research Center (BCRC) in Taiwan and maintained in Dulbecco's modified Eagle's medium supplemented with 10% fetal bovine serum at 37°C in a humidified atmosphere of 5% CO_2_. Cells in 24-well plates (2 × 10^5^ cells per well) were treated with LPS (100 ng mL^−1^) and test compounds (RMRE, lovastatin, citrinin, monascin and ankaflavin).

### 2.6. Nitrite and PGE_2_ Production Determination

Nitrite levels in cell culture supernatants were determined by Griess reaction. The supernatants (0.05 mL) were simultaneously treated with 0.05 mL Griess reagent A (1% sulfanilamide in 2.5% H_3_PO_4_) and 0.05 mL Griess reagent B (0.1% *N*-1-naphthyl ethylene diaminedihydrochloride in 2.5% H_3_PO_4_) for 10 min at room temperature. NaNO_2_ was used to generate a standard curve; nitrite production was measured by a spectrophotometer at 570 nm [26]. PGE_2_ concentrations were determined by a prostaglandin E_2_ immunoassay kit [25].

### 2.7. Western Blot Analysis

RAW264.7 cells (2 mL, 4 × 10^6^ cells mL^−1^), grown in a 6-cm dish, were incubated with or without LPS in the absence or presence of the test compounds for 24 h, respectively. Cells were lysed with ice-cold PBS containing 1% nonidet P-40, 1 mM of phenylmethylsulfonyl fluoride, 10 mg mL^−1^ of aprotinin, 50 mM of sodium fluoride and 2 mM of sodium orthovanadate (Sigma-Aldrich). Protein lysates (30 *μ*g) were separated using 10% SDS-polyacrylamide gel electrophoresis and transferred to a polyvinylidene difluoride membrane (Millipore, Billerica, MA, USA). After they had been blocked with 10% milk in TBS-T (10 mM Tris (pH 7.6), 150 mM NaCl and 0.05% Tween 20), the membranes were incubated with appropriate antibodies containing primary antibody and anti-rabbit or anti-mouse secondary horseradish peroxidase antibodies (Sigma-Aldrich), iNOS and *β*-actin antibody (Santa Cruz Biotechnology, Santa Cruz, CA, USA) and COX-2 antibody (Transduction Laboratories Biotechnology, Lake Placid, NY, USA). Subsequently, the blots were visualized using a chemiluminescence kit (PerkinElmer, Boston, MA, USA). *β*-actin was set as an internal control. The optical densities of the bands were determined using UVP autochemi image system (UVP Inc., Upland, CA, USA).

### 2.8. Data Analysis

Results are presented as mean ± SD and statistical significance was determined by Student's *t*-test. *P* < .05 was considered as indicating statistical significance.

## 3. Results

### 3.1. RMRE Reduces Carcinogenesis in DMBA-Induced Oral Tumor Model

As shown in Table 1, DMBA treatment in hamster cheek pouch induced tumor formation, whereas mineral oil treatment as a control group did not induce the formation of oral tumor. Treatment with 6% celecoxib grossly reduced the burden of tumor. The RMRE group significantly decreased tumor burden (*P* < .05). On the other hand, RMRE treatment exerted a better anti-tumor activity than that of lovastatin treatment alone.

### 3.2. RMRE Decreased ROS, and PGE_2_ Levels in DMBA-Induced Oral Tumor Model

**Figure 2:**
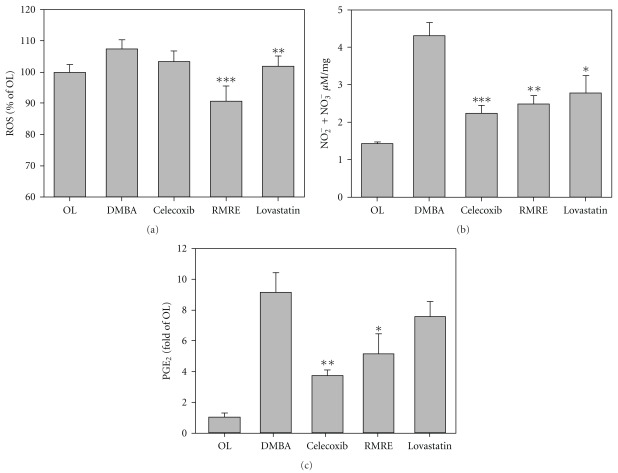
RMRE decreased ROS, NO_2_^−^NO_3_^−^ and PGE_2_ levels in experimental hamsters. The homogenates of buccal pouch was measured levels of ROS (a), NO_2_^−^/NO_3_^−^ (b) and PGE_2_ (c). Results are expressed as the mean ± SD, *n* = 5. *P < .05, **P < .01, ***P < .001 versus the DMBA treatment group.

As shown as Figure 2, the levels of ROS, NO_2_^−^/NO_3_^−^ and PGE_2_ in the cheek pouch of hamsters in DMBA-induced oral mucositis were significantly increased as compared with the control group. RMRE treatment significantly reduced DMBA-induced increment in ROS, NO_2_^−^/NO_3_^−^ and PGE_2_ levels than those of the lovastain-treated group. The lower levels of NO_2_^−^/NO_3_^−^ and PGE_2_ were also observed in the celecoxib group.

### 3.3. Inhibition of LPS-Induced Nitrite Production by RMRE in RAW264.7 Cells

**Figure 3:**
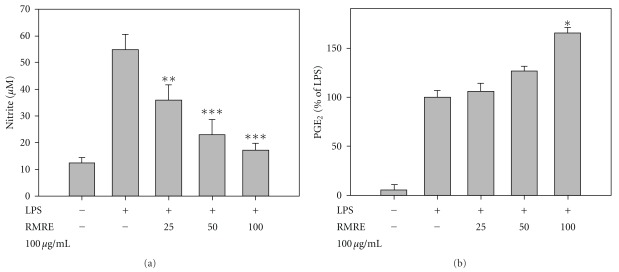
The effects of RMRE on LPS-induced nitrite and PGE_2_ generation in RAW264.7 cells. Various concentrations of RMRE affected the LPS-induced levels of nitrite (a) and PGE_2_ (b), determined at 48 h. Results are expressed as the mean ± SD for at least three independent experiments. **P < .01, ***P < .001 versus the LPS treatment group.

RMRE treatment has no effect on cell viability of RAW264.7 cells, determined by MTT assay (data not shown). As shown in Figure 3, RMRE inhibited nitrite production in a dose-dependent manner with an IC_50_ of 40.3 *μ*g mL^−1^ whereas RMRE had no effect on LPS-induced PGE_2_ production in RAW264.7 cells.

### 3.4. RMRE Inhibited LPS-Induced iNOS Protein Expression

**Figure 4:**
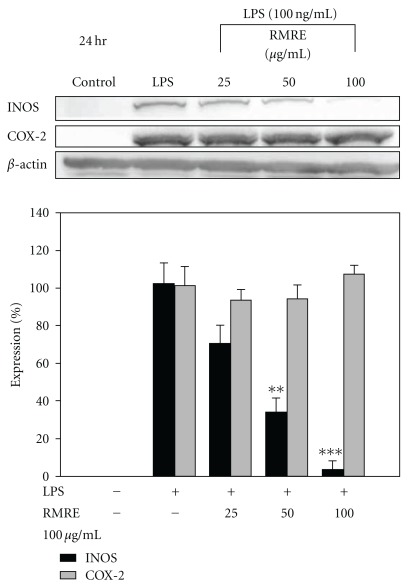
The effects of RMRE on iNOS/COX-2 expression in RAW264.7 cells. Equal amounts of total proteins (80 *μ*g per lane) were subjected to 10% SDS-PAGE and expression of iNOS, COX-2 and *β*-actin was detected by western blotting analysis. *β*-actin was used as an internal control. Results are expressed as the mean ± SD for at least three independent experiments. Treatment of RMRE for 24 h decreased LPS-induced iNOS expression. **P < .01, ***P < .001 versus the LPS treatment group.

We detected LPS-Induced iNOS and COX-2 protein expression after RMRE treatment for 24 h in RAW264.7 cells. As shown in Figure 4, LPS treatment elevated the expression of iNOS and COX-2, and RMRE significantly reduced the protein expression of iNOS (*P* < .001), but had no effect on COX-2 levels.

### 3.5. Inhibitory Effects of Bioactive Compounds of RMRE on LPS-Induced Nitrite and PGE Generation in RAW264.7 Cells

**Figure 5:**
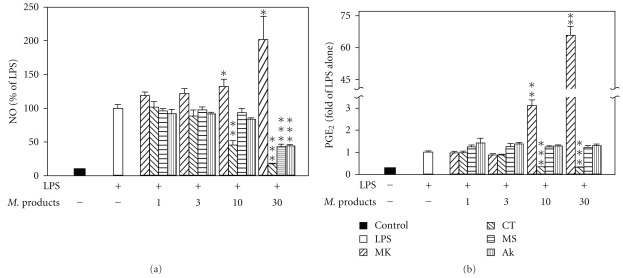
Inhibitory effects of bioactive compounds on LPS-induced nitrite and PGE_2_ generation in RAW264.7 cells. Treatment of various concentrations of different compounds (lovastatin: MK, monascin: MS, ankaflavin: AK and citrinin: CT) for 48 h decreased LPS-induced nitrite (a) and PGE_2_ (b) generation. Results are expressed as the mean ± SD for at least three independent experiments. *P < .05, **P < .01, ***P < .001 versus the LPS treatment group.

As shown in Figure 5, we found that 30 *μ*M lovastatin treatment exerts no inhibition activity on LPS-induced nitrite and PGE_2_ generation because of the decrement in cell viability (data not shown). On the other hand, both monascin and ankaflavin in 30 *μ*M inhibited nitrite production and had no effect on PGE_2_ expression. Moreover, citrinin suppressed LPS-induced nitrite and PGE_2_ production in a dose-dependent manner. High concentrations of citrinin, monascin or ankaflavin, except lovastatin, may not lead to cell death.

## 4. Discussion

Nowadays, complementary and alternative medicine (CAM) is widely available throughout the world. Cancer patients are exposed to CAM ranging from health supplements to traditional forms of medicine like traditional Chinese medicine (TCM) and traditional Indian medicine. In the past studies, the aqueous extract of neem which grows throughout India has been found to possess anti-inflammatory and potent chemopreventive activity [27].

Several studies have been demonstrated that red mold rice has an anti-tumor effect; however, the possible mechanisms and the main components of the red mold rice to exert the effect are still unclear. In this study, we found that RMRE reduces ROS, NO_2_^−^/NO_3_^−^ and PGE_2_ levels in DMBA-induced oral tumor model (*P* < .001). In addition, RMRE significantly inhibited LPS-induced nitrite levels in RAW264.7 cells than monacolin K, monascin, ankaflavin and citrinin (*P *< .05) and this effect might play a role in the prevention of oral tumor.

Celecoxib has a chemopreventive action against DMBA-induced cheek pouch carcinogenesis [28]. In our result, RMRE and 6% celecoxib grossly but significantly inhibited the tumor formation (Table 1). However, we found that the lovastatin and celecoxib-treated animals had lower number of tumors but the volume was grossly greater than control animals. This effect might be because of the anti-inflammation action of the drugs and this effect further prevents from the cell mutation and tumor formation. However, these drugs might have less therapeutic effect. In addition, this effect may be because of the large variation of the incidence of tumor formation in DMBA-induced oral tumor model or the dosage of RMRE treatment.

**Figure 6:**
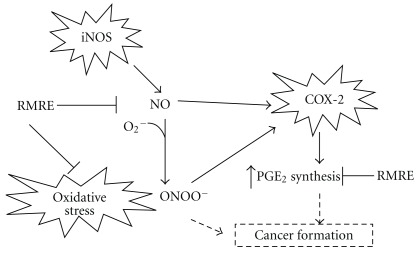
The correlations of RMRE inhibiting ROS, NO and PGE_2_ levels in DMBA-induced oral tumor model. iNOS expression induced NO generation and high NO levels might lead to oral squamous cell carcinoma development. On the other hand, COX-2 expression promoted PGE_2_ synthesis, and high PGE_2_ expression was increased in oral SCC tissue as compared with normal tissue. In this study, RMRE treatment had pharmacological inhibition of ROS, NO and PGE_2_ *in vivo*. It might be a possible strategy for oral cancer prevention.

Studies indicated that elevated expression of inducible nitric oxide synthase (iNOS) was observed in both human oral carcinogenesis and chemically induced oral carcinogenesis in rodents [29, 30]. Increased iNOS expression and the generation of high NO levels might lead to oral squamous cell carcinoma development [31], implying that pharmacological inhibition of iNOS and NO might be a possible strategy for oral cancer prevention. Moreover, COX-2 was shown to be over-expressed in both premalignant and malignant lesions of the oral cavity with increased expression from hyperplasia to dysplasia and squamous cell carcinogenesis (SCC) [32]. Also, the PGE_2_ level was increased in oral SCC tissue as compared with normal tissue [33]. In this study, we found that RMRE significantly inhibited DMBA-induced ROS, NO and PGE_2_ levels in homogenates of oral tissue (*P* < .001). These results imply that RMRE might inhibit DMBA-induced oral tumor carcinogenesis through anti-inflammatory and antioxidant effects as shown in Figure 6.

Previous studies indicated that monacolin K which is one of the main active components in red mold rice at 30 *μ*M suppressed LPS-induced nitrite generation; however, the expression of COX-2 was promoted to induce PGE_2_ generation in RAW264.7 macrophages [34–36]. This result is inconsistent with our results in animals. In our study, RMRE has no effect on cell visibility in used concentrations and the inconsistencies of the results *in vivo* and *in vitro* might partly be because of the RMRE metabolites produced in the cell and not secreted to the medium. In addition, statins directly reduced prostaglandin E2 (PGE_2_) release [37] and NO production [38] *in vivo*, whereas statins might decrease the production of NO through an anti-inflammation effect indirectly. These effects might be applied to explain the inconsistencies in *in vivo* and *in vitro* results. We further investigated the main components in red mold rice to exert the anti-tumor effect. Thus, we used HPLC to determine the composition of RMRE, and we found that it contains 1.26 *μ*M monacolin K in 100 *μ*g mL^−1^ RMRE. However, it exerts a significant effect on the inhibition of nitrite production (*P* < .001). RMRE exerted a more effective action as compared with any one of the single components. This result reveals that RMRE might contain other compounds that inhibit nitrite production or the synergic effect between the components. We further tested 1, 3, 10 and 30 *μ*M of other main components of RMRE, such as monascin, ankaflavin, citrinin in LPS-induced RAW264.7 cells. It was reported that oral administration of monascin inhibited the carcinogenesis of skin cancer, initiated by peroxynitrite or ultraviolet light and after the promotion of 12-*O*-tetradecanoyl-phorbol-13-acetate (TPA) [9]. In addition, ankaflavin showed selective cytotoxicity to cancer cell lines by an apoptosis-related mechanism [39]. As shown in 4, monascin or ankaflavin at 30 *μ*M inhibits LPS-induced nitrite generation but shows no effects on PGE_2_ production (*P* < .001). Ten micromolar citrinin had an inhibition activity to decrease LPS-induced nitrite (*P* < .05) or PGE_2_ generation (*P* < .001). However, 100 *μ*g mL^−1^ of RMRE contains only 3.58 *μ*M monascin, 1.07 *μ*M ankaflavin and 0.01 *μ*M citrinin. The concentrations of those components are much lower than previous studies in the inhibition of nitrite generation. Thus, RMRE might inhibit LPS-induced nitrite generation through other unidentified compounds. Other studies also suggested that the matrix effects of red mold rice beyond monacolin K alone might be active in inhibiting cancer growth [40, 41]. Taken together, RMRE exerts a chemoprevention activity in the inhibition of inflammation and oxidation which is possible for oral cancer prevention. In addition, our study showed that the matrix effects of RMRE beyond monacolin K alone may be active in mitigates oral carcinogenesis.

## Figures and Tables

**Table 1:** Incidence of oral neoplasm in control and experiment animal groups (*n* = 5).

Group	Treatment	No. of tumor	Mean tumor volume (mm^3^)	Tumor burden (mm^3^)
D	DMBA 6 weeks alone	18	26.92 ± 20.11	92.86 ± 63.68
A	DMBA 6 weeks + 6% celecoxib	9	48.57 ± 20.86	72.85 ± 28.59
B	DMBA 6 weeks + RMRE	10	23.74 ± 21.34	35.76 ± 18.49*
C	DMBA 6 weeks + Lovastatin	10	42.25 ± 25.42	99.42 ± 98.71
OL	Oil 14 weeks	0	—	—

*Significantly different when compared with group D (*P* < .05) by Student's *t*-test.
